# Pre-Dialysis Visits to a Nephrology Department and Major Cardiovascular Events in Patients Undergoing Dialysis

**DOI:** 10.1371/journal.pone.0147508

**Published:** 2016-02-22

**Authors:** Chih-Yuan Huang, Chia-Wen Hsu, Chi-Rou Chuang, Ching-Chih Lee

**Affiliations:** 1 Division of Nephrology, Department of Internal Medicine, Ditmanson Medical Foundation Chia-Yi Christian Hospital, Chia-Yi, Taiwan; 2 Department of Medical Research, Dalin Tzu Chi Hospital, Buddhist Tzu Chi Medical Foundation, Chia-Yi, Taiwan; 3 Department of Obstetrics and Gynecology, Dalin Tzu Chi Hospital, Buddhist Tzu Chi Medical Foundation, Chia-yi, Taiwan; 4 Department of Otorhinolaryngology, Head and Neck Surgery, Kaohsiung Veterans General Hospital, Kaohsiung, Taiwan; 5 School of Medicine, National Defense Medical Center, Taipei, Taiwan; The University of Tokyo, JAPAN

## Abstract

**Background and Objectives:**

Pre-dialysis care by a nephrology out-patient department (OPD) may affect the outcomes of patients who ultimately undergo maintenance dialysis. This study examined the effect of pre-dialysis care by a nephrology OPD on the incidence of one-year major cardiovascular events after initiation of dialysis.

**Design, Setting Participants, & Measurements:**

The study consisted of Taiwanese patients with chronic kidney disease (CKD) who commenced dialysis from 2006 to 2008. The number of nephrology OPD visits during the critical care period (within 6 months of initiation of dialysis) and the early care period (6–36 months before initiation of dialysis) were analyzed. The primary outcome measure was one-year major cardiovascular events.

**Results:**

A total of 1191 CKD patients who initiated dialysis from 2006 to 2008 were included. Binary logistic regression showed that patients with ≧3 visits during the critical care period and those with ≧11 visits during the early care period had fewer composite major cardiovascular events than those with 0 visits. Patients with early referral are less likely to experience composite major cardiovascular events than those with late referral, with aOR 0.574 (95% CI = 0.43–0.77, P<0.001). Patients with both ≧3 visits during critical care period and ≧11 visits during early care period were less likely to experience composite major cardiovascular events (aOR = 0.25, 95% CI = 0.16–0.39, P < 0.001).

**Conclusions:**

Patients with adequate pre-dialysis nephrology OPD visits, not just early referral, may had fewer one-year composite major cardiovascular events after initiation of dialysis. This information may be important to medical care providers and public health policy makers in their efforts to improve the well-being of CKD patients.

## Introduction

Chronic kidney disease (CKD), which has a prevalence of 11.93% in Taiwan [1], is a worldwide public health issue because it is associated with high mortality and morbidity. Not all CKD patients are first recognized by nephrologists. A recent study indicated that primary care physicians (PCPs) recognize and recommend specialist care for CKD patients less often than nephrologists, and that these 2 groups also differ in their clinical evaluations and expectations for referral [2]. The situation is similar among different internal medicine residents [3].

Numerous studies indicated the importance of early referral to a nephrologist. In particular, these studies reported an association between late referral for CKD care and adverse outcomes, such as choice of dialysis modality [4, 5], inpatient start or emergent initiation of renal replacement therapy [4, 6], use of a hemodialysis catheter [6, 7], and mortality [6, 8, 9]. Other studies reported that the advantages of early referral are provision of appropriate therapy based on diagnosis, slowing or arresting the progression of CKD, prevention and management of comorbid conditions and CKD-specific complications, planning and preparation for maintenance dialysis, and psychosocial support[10].

Previous studies have somewhat arbitrarily defined early and late referral by the date of the first nephrology outpatient visit, before or after anywhere from 1 to 12 months before initiation of maintenance dialysis [11]. The effects of multiple earlier nephrology outpatient department (OPD) visits are rarely considered. Therefore some studies demonstrated relatively high mortality and suboptimal initiation of dialysis in patients who receive early referral [12, 13]. Only a few studies have compared the effect of multiple early nephrology OPD visits within 6 or 12 months before initiation of dialysis [14–16]. Recently, Singhal et al. showed that the total nephrology OPD visits within 1–6 months and 1–36 months before initiation of dialysis were associated with one-year mortality after initiation of renal replacement therapy (RRT) [17]. However, they did not separately evaluate myocardial and stroke events in these patients.

Studies that focus on pre-dialysis care should exclude patients with acute kidney injury (AKI) who do not recover and therefore require long-term RRT[17]. These patients might not have had CKD before development of AKI, and also did not receive adequate preparation or education. Moreover, the pathogenesis of AKI is very different from that of CKD, and inclusion of AKI patients may therefore bias the results. Several criteria could be potentially used to identify patients with AKI, such as no prior nephrology visits, initiation of continuous RRT, or eventual recovery of renal function.

The aim of this study was to analyze the cumulative effect of nephrology OPD visits on the occurrence of major cardiovascular events within 1 year after initiation of dialysis. We also investigated variables associated with late referral to nephrologists.

## Materials and Methods

### Ethics statements

This study was approved by the Institutional Review Board of Buddhist Dalin Tzu Chi Hospital, Buddhist Tzu Chi Medical Foundation, Taiwan. Data in the National Health Insurance Research Database (NHIRD) that could be used to identify patients or care providers, including medical institutions and physicians, was anonymized before being sent to the National Health Research Institutes for database construction and was further anonymized before release to researchers. The review board thus stated that written consent from patients was not required. We also adhered to the Declaration of Helsinki (Available at: http://www.wma.net/en/30publications/10policies/b3/, Access date: August 1, 2015.)

### Database

The data for this study were collected from the files of Taiwan’s NHIRD for the years 2002 to 2009. This dataset is organized and managed by Taiwan’s National Health Research Institutes from data collected by Taiwan’s National Health Insurance Program, which has been in place since 1995. The program covers approximately 99% of the residents in Taiwan and has contracts with 97% of the medical providers. A growing body of evidence has confirmed the validity of the NHIRD [18, 19].

### Patients and study design

Our study cohort consisted of patients with diagnoses of CKD who began dialysis from 2006 to 2008. S1 Fig showed patient disposition. CKD was identified by the International Classification of Diseases, Ninth Revision, Clinical Modification [ICD-9-CM] codes 585 and 586. The treatment codes were “D8” for hemodialysis and “D9” for peritoneal dialysis, so we used these codes to further characterize patients undergoing regular dialysis. All patients were at least 18 years-old. We excluded patients who began regular dialysis before 2006, those who underwent kidney transplantation (coded as “V42.0” in the Catastrophic Illness Dataset of NHIRD), those with AKI that required dialysis, and those who had a dialysis-free period longer than 60 days after a series of dialysis records [17].

### Measurements

#### Initiation of dialysis

We defined the first date of a series of regular dialysis treatments in OPD as the date of dialysis initiation. If an admission with a dialysis treatment code preceded this OPD event, the date of this admission was regarded as the initiation date. In the NHIRD, treatments and procedures during admission were not dated.

#### Critical care period, early care period, early referral, and late referral

The critical care period was defined as the time within 6 months before initiation of dialysis [17], and the early care period was defined as the time 6 to 36 months before initiation of dialysis. Patients with any nephrology OPD visit during early care periods were defined as early referral patients (6 to 36 months before initiation of dialysis). And those without any nephrology OPD visit during early care periods were defined as late referral patients.

#### Composite major cardiovascular events

Kip KE et al did literature review and found that there was no consensus definition of major adverse cardiovascular events, usually abbreviated as MACE. Varying definitions of composite end points can lead to substantially different results and conclusions [20]. This study used the nomenclature of composite major cardiovascular events, which was consisted of all-cause mortality, acute myocardial infarction and stroke, to avoid confusion.

#### Other variables

Patient demographics included age, gender, socioeconomic status (individual income), urbanization, initial dialysis modality, and disease severity. Disease severity was estimated with the Deyo adaptation of the Charlson Comorbidities Index Score (CCIS), which was derived from inpatient and outpatient diagnoses in the last six months of life [21]. CKD was excluded in calculation of the CCIS.

### Statistical analysis

The SAS statistical package (version 9.2; SAS Institute, Inc., Cary, N.C.) and SPSS (version 15, SPSS Inc., Chicago, IL, USA) was used to analyze the NHIRD dataset. A *p*-value less than 0.05 was used to define statistical significance. Binary logistic regression was used to evaluate the effect of demographic characteristics on nephrology OPD visits in the critical care period (within 6 months before the initiation of dialysis) and the early care period (6–36 months before initiation of dialysis). Binary logistic regression was also used to assess the effect of variables on 1- year composite major cardiovascular events after initiation of dialysis by calculation of adjusted ORs (aORs) and 95% confidence intervals (95% CIs). We also analyzed the combined effect of nephrology OPD visits in the critical care period and the early care period on 1-year composite major cardiovascular events and its components after initiation of dialysis by binary logistic regression.

## Results

This study initially enrolled 4324 Taiwanese patients with CKD (ICD-9-CM codes “585” or “586”) who started maintenance renal replacement therapy (hemodialysis or peritoneal dialysis) from 2006 to 2008. After exclusion of ineligible patients according to pre-established criteria (S1 Fig), there were 1191 eligible CKD patients.

Table 1 shows the basic characteristics of these CKD patients. Analysis of the critical care period indicated that 363 patients had 0 visits, 155 patients had 1–2 visits, and 673 patients had 3 or more visits to the nephrology OPD. Analysis of the early care period indicated that 490 patients had 0 visits, 194 patients had 1–5 visits, 113 patients had 6–10 visits, and 394 patients had 11 or more visits to the nephrology OPD. 346 CKD patients had both ≧3 nephrology OPD visits in critical care period and ≧11 nephrology OPD visits in early care period.

**Table 1:** Baseline characteristics of patients with chronic kidney disease who had different numbers of nephrology out-patient department visits during the critical care period (within 6 months before initiation of dialysis) and the early care period (6–36 months before initiation of dialysis).

	All patients *n* = 1191	Critical care period				Early care period				
		0 visit *n* = 363	1–2 visits *n* = 155	≧3 visits *n* = 673	P value	0 visit *n* = 490	1–5 visits *n* = 194	6–10 visits *n* = 113	≧11 visits *n* = 394	P value
**Median age, years (IQR)**	67(56,76)	68(56,78)	66(55,75)	67(57,76)	0.616	66(55,75)	57(57,75)	57(59,77)	68(56,77)	0.317
**Age≥75 years (%)**	348(29.2)	114(31.4)	42(27.1)	192(28.5)	0.514	135(27.6)	51(26.3)	36(31.9)	126(32.0)	0.348
**Sex**					0.133					0.251
Female (%)	594(49.9)	175(48.2)	68(43.9)	351(52.2)		229(46.7)	98(50.5)	63(55.8)	204(51.8)	
Male (%)	597(50.1)	188(51.8)	87(56.1)	322(47.8)		261(53.3)	96(49.5)	50(44.2)	190(48.2)	
**Socioeconomic status**					0.413					0.811
Low (%)	612(51.4)	180(49.6)	75(48.4)	357(53.0)		249(50.8)	95(49.0)	60(53.1)	208(52.8)	
Mod./High (%)	579 (48.6)	183(50.4)	80(51.6)	316(47.0)		241(49.2)	99(51.0)	53(46.9)	186(47.2)	
**Urbanization**					0.601					0.656
Urban/Suburban (%)	879(73.8)	273(75.2)	110(71.0)	496(73.7)		359(73.3)	141(72.7)	80(70.8)	299(75.9)	
Rural(%)	312(26.2)	90(24.8)	45(29.0)	177(26.3)		131(26.7)	53(27.3)	33(29.2)	95(24.1)	
**CCIS count, median (IQR)**	3(1,4)	2(1,4)	3(1,4)	3(2,4)	0.015	2(1,4)	3(2,4)	3(1,4)	2(1,4)	0.023
**Comorbidities**										
Hyperlipidemia	64(5.4)	13(3.6)	7(4.5)	44(6.5)	0.116	29(5.9)	13(6.7)	2(1.8)	20(5.1)	0.271
Hypertension	824(69.2)	223(61.4)	115(74.2)	486(72.2)	0.001	331(67.6)	139(71.6)	90(79.6)	264(67.0)	0.049
**Initial RRT modality**					0.004					0.018
HD, %	1065(89.4)	339(93.4)	141(91.0)	585(86.9)		448(91.4)	179(92.3)	101(89.4)	337(85.5)	
PD, %	126(10.6)	24(6.6)	14(9.0)	88(13.1)		42(8.6)	15(7.7)	12(10.6)	57(14.5)	
**Early care period**										
0 visit, %	490(41.1)	261(71.9)	92(59.4)	137(20.4)	<0.001					
≦5 visits, %	194(16.3)	59(16.3)	32(20.6)	103(15.3)						
6–10 visits, %	113(9.5)	15(4.1)	11(7.1)	87(12.9)						
≧11 visits, %	394(33.1)	28(7.7)	20(12.9)	346(51.4)						
**Early Referral** (by traditional definition, any visit in early care period)	701(58.9)	102(28.1)	63(40.6)	536(79.6)	<0.001		194(100)	113(100)	394(100)	<0.001
**Late Referral** (by traditional definition, no visit in early care period)	490(41.1)	261(71.9)	92(59.4)	137(20.4)		490(100)				

Abbreviations: IQR, interquartile range; CCIS, Charlson Comorbidity Index; CKD, chronic kidney disease; HD, hemodialysis, PD, peritoneal diualysis; RRT, renal replacement therapy.

In Table 2, binary logistic regression analysis of variables associated with nephrology OPD visits showed that, compared with those without cerebrovascular disease, CKD patients with cerebrovascular disease were more likely to have fewer than 3 nephrology OPD visits (OR = 0.53, 95% CI = 0.37–0.75, P < 0.001) during the critical care period. In contrast, CKD patients with hypertension were more likely to have 3 or more nephrology OPD visits during this period (OR = 1.37, 95% CI = 1.05–1.78, P = 0.020). And during the early care period, CKD patients with congestive heart failure (OR = 0.62, 95% CI = 0.46–0.83, P = 0.002) and cardiovascular disease (OR = 0.57, 95% CI = 0.39–0.81, P = 0.002) were less likely to have 11 or more nephrology OPD visits.

**Table 2:** Mutivariable analysis of the association of demographic and clinical characteristics of patients with three or more nephrology OPD visits during the critical care period (within 6 months before initiation of dialysis) and eleven or more nephrology OPD visits during the early care period (6–36 months before initiation of dialysis).

	Three or more nephrology OPD visits during the critical care period			Eleven or more nephrology OPD visits during the early care period		
	Odds Ratio	95% CI	P	Odds Ratio	95% CI	P
**Age**	1.01	(1.00–1.01)	0.198	1.01	(1.00–1.02)	0.065
**Sex**						
Female	1			1		
Male	0.83	(0.65–1.05)	0.118	0.81	(0.64–1.03)	0.083
**Comorbidities**						
Myocardial infarction	0.81	(0.46–1.40)	0.446	0.79	(0.45–1.40)	0.418
Congestive Heart Failure	0.83	(0.62–1.12)	0.228	0.62	(0.46–0.83)	0.002
Cerebrovascular Disease	0.53	(0.37–0.75)	<0.001	0.57	(0.39–0.81)	0.002
Chronic obstructive pulmonary disease	0.80	(0.53–1.20)	0.277	1.21	(0.80–1.83)	0.357
Peptic Ulcer Disease	1.05	(0.75–1.46)	0.788	0.97	(0.70–1.35)	0.852
Diabetes Mellitus	1.23	(0.96–1.57)	0.107	0.91	(0.71–1.17)	0.465
Liver Disease	0.80	(0.45–1.40)	0.431	1.01	(0.57–1.79)	0.970
Hyperlipidemia	1.61	(0.93–2.80)	0.092	1.21	(0.72–2.03)	0.474
Hypertension	1.37	(1.05–1.78)	0.020	1.18	(0.91–1.54)	0.213
**Socioeconomic status**						
Low	1			1		
Moderate/ High	0.83	(0.65–1.05)	0.121	0.84	(0.67–1.07)	0.160
**Urbanization**						
Urban/ Suburban	1			1		
Rural	1.08	(0.82–1.40)	0.623	0.91	(0.70–1.20)	0.514

Abbreviations: CI, confidence interval; OPD, outpatient department.

**Fig 1:**
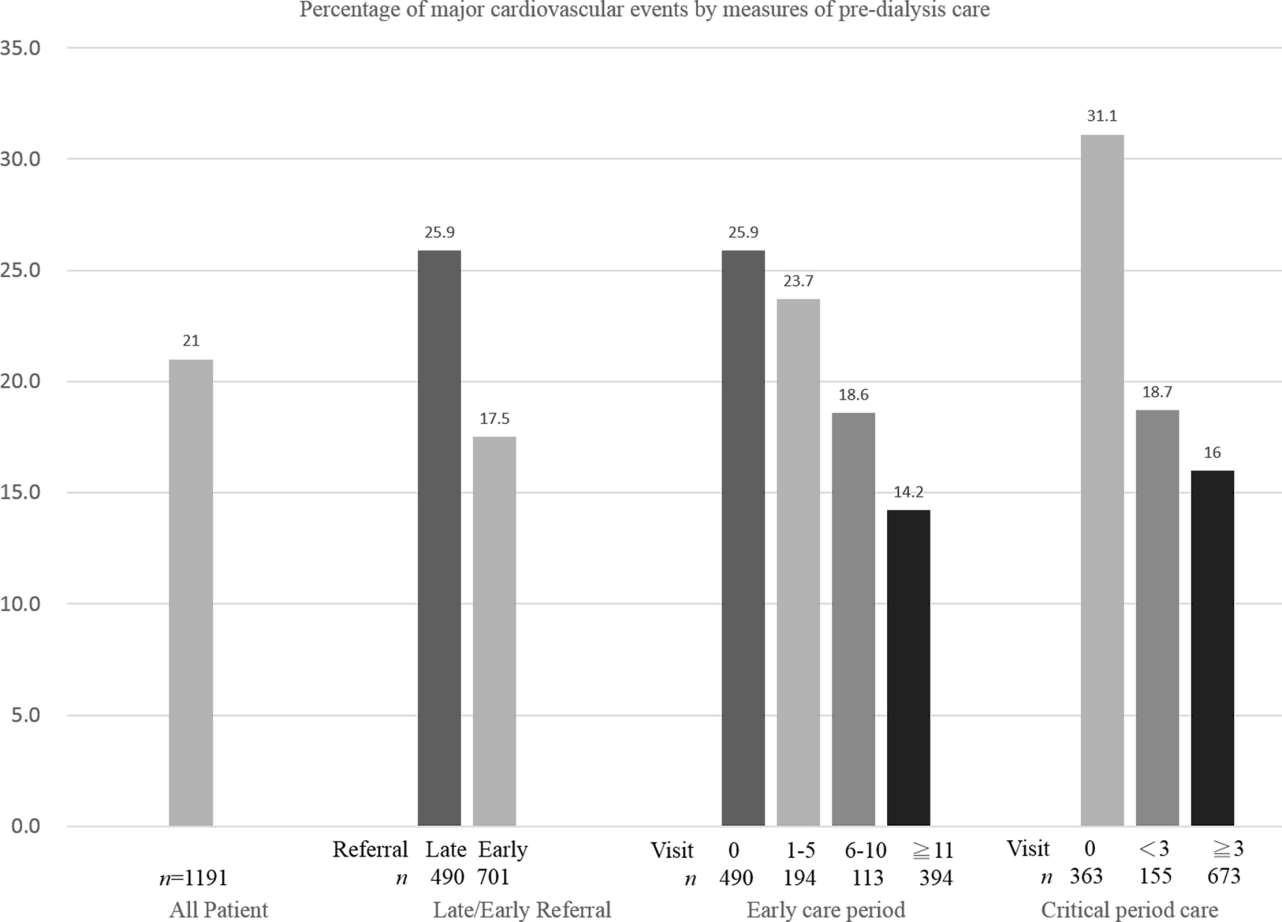
Percentage of major cardiovascular events by measures of pre-dialysis care. Percentage of patients with chronic kidney disease who had a major cardiovascular event after initiation of dialysis according to the number of nephrology outpatient department visits during the critical care period (within 6 months before initiation of dialysis) and the early care period (6–36 months before initiation of dialysis) and early or late referral (within 6 months).

In Table 3, we analyzed the incidence of composite major cardiovascular events in the one year after initiation of dialysis. The overall incidence of composite major cardiovascular events were 31.1%, 18.7%, and 16.0% in patients with 0, 1–2, and 3 or more nephrology OPD visits during the critical care period, respectively. The overall incidence of composite major cardiovascular events were 25.9%, 23.7%, 18.6%, and 14.2% in patients with 0, 1–5, 6–10, and 11 or more nephrology OPD visits during the early care period, respectively. The incidences of stroke and all-cause mortality were lower for those with three or more nephrology OPD visits in the critical care and early care periods. Fig 1 shows the overall percentage of CKD patients with composite major cardiovascular events, the percentage of these events among those with early/late referral, and the percentage of these events among patients with different numbers of nephrology OPD visits during the critical care and the early care periods.

**Table 3:** One-year incidence rates of major cardiovascular events in patients receiving dialysis according to the number of nephrology out-patient department visits during the critical care period (within 6 months before initiation of dialysis) and the early care period (6–36 months before initiation of dialysis).

Variables	Stroke (%)	P	AMI (%)	P	Mortality (%)	P	MACE (%)	P
**Critical care period**		<0.001		0.157		<0.001		<0.001
0 visit (*n* = 363)	51(14.0)		8(2.2)		74(20.4)		113(31.1)	
1–2 visits (*n* = 155)	11(7.1)		0(0.0)		23(14.8)		29(18.7)	
≧3 visits (*n* = 673)	45(6.7)		16(2.4)		63(9.3)		108(16.0)	
**Early care period**		0.008		0.804		0.030		<0.001
0 visit (*n* = 490)	54(11.0)		12(2.4)		80(16.3)		127(25.9)	
1–4 visits (*n* = 194)	23(11.9)		4(2.1)		29(14.9)		46(23.7)	
6–10 visits (*n* = 113)	10(8.8)		2(1.8)		12(10.6)		21(18.6)	
≧11 visits (*n* = 394)	20(5.1)		6(1.5)		39(9.9)		56(14.2)	
**Early Referral** (*n* = 701)	53(7.6)	0.050	12(1.7)	0.406	80(11.4)	0.016	123(17.5)	<0.001
**Late Referral** (*n* = 490)	54(11.0)		12(2.4)		80(16.3)		127(25.9)	

Abbreviations: AMI, acute myocardial infarction; MACE, major cardiovascular event.

Table 4 shows the results of binary logistic regression analysis of variables associated with composite major cardiovascular events at 1 year after initiation of dialysis. Analysis of the critical care period indicated that patients with 1–2 visits (aOR = 0.47, 95% CI = 0.28–0.77, P = 0.003) and patients with 3 or more visits (aOR = 0.47, 95% CI = 0.33–0.69, P <0.001) were less likely to experience a composite major cardiovascular event than those with 0 visits. Analysis of the early care period indicated that patients with 11 or more nephrology OPD visits (aOR = 0.62, 95% CI = 0.41–0.96, P = 0.032) were less likely to experience a composite major cardiovascular event than those with 0 visits. Patients with early referral are less likely to experience composite major cardiovascular events than those with late referral, with an aOR 0.574 (95% CI = 0.43–0.77, P<0.001). Further analysis indicated that model discrimination was good (c-statistic = 0.742). We also investigated variables associated with stroke, acute myocardial infarction, and all-cause mortality. Patients with 3 or more visits during the critical care period were less likely to experience a stroke (aOR = 0.49, 95% CI = 0.30–0.82, P = 0.006) than those with 0 visits. Moreover, patients with 3 or more visits during the critical care period had lower all-cause mortality (aOR = 0.44, 95% CI = 0.28–0.69, P < 0.001) than those with 0 visits. Table 4 also showed the results of the combined effect of nephrology OPD visits in both critical care period and early care period on 1-year composite major cardiovascular event rates and its components after initiation of hemodialysis. CKD patients with both 3 or more nephrology OPD visits in critical care period and eleven or more nephrology OPD visits in early care period were less likely to experience composite major cardiovascular events than those with 0 visits in both critical care period and early care period (aOR = 0.25, 95% CI = 0.16–0.39, P < 0.001). Similar effect was observed in CKD patients who experience stroke (aOR = 0.26, 95% CI = 0.14–0.49, P < 0.001) and all-cause mortality (aOR = 0.30, 95% CI = 0.17–0.50, P < 0.001) in S1 Table.

**Table 4:** Mutivariable analysis of the association of visits during the critical care period (within 6 months before initiation of dialysis) and the early care period (6–36 months before initiation of dialysis) with severe vascular events at 1 year after initiation of renal replacement therapy.

**Variable**	**Stroke**			**AMI**			**Mortality**			**Composite major cardiovascular events**		
	aOR*	**95% CI**	**P**	aOR*	**95% CI**	**P**	aOR*	**95% CI**	**P**	aOR*	**95% CI**	**P**
**Critical care period**												
0 visit	1			1			1			1		
1–2 visits	0.42	(0.21–0.86)	0.017	--	--		0.69	(0.40–1.19)	0.189	0.47	(0.28–0.77)	0.003
≧3 visits	0.49	(0.30–0.82)	0.006	1.72	(0.64–4.62)	0.283	0.44	(0.28–0.69)	<0.001	0.47	(0.32–0.69)	<0.001
**Early care period**												
0 visit	1			1			1			1		
1–4 visits	1.22	(0.71–2.12)	0.811	0.71	(0.21–2.32)	0.572	1.05	(0.64–1.72)	0.845	0.97	(0.64–1.47)	0.897
6–10 visits	0.98	(0.45–2.12)	0.539	0.48	(0.09–2.36)	0.368	0.90	(0.44–1.82)	0.771	0.82	(0.46–1.45)	0.498
≧11 visits	0.58	(0.31–1.07)	0.076	0.41	(0.13–1.26)	0.123	0.85	(0.52–1.41)	0.549	0.62	(0.41–0.96)	0.032
c-statistic	0.725			0.767			0.755			0.740		
Hosmer–Lemeshow goodness-of-fit statistic	0.218			0.552			0.885			0.845		
**Early referral**	0.637	(0.42–0.96)	0.030	0.704	(0.31–1.59)	0.399	0.650	(0.46–0.92)	0.016	0.574	(0.43–0.77)	<0.001
**Late referral**	1			1			1			1		
	Critical care period (aOR* **of** composite major cardiovascular events)**											
	**0 visit *n* = 363**	**95% CI**	**P**		**1–2 visits *n* = 155**	**95% CI**		**P**	**≧3 visits *n* = 673**		**95% CI**	**P**
**Early care period**												
0 visit, %	1				0.42	(0.22–0.79)		0.007	0.43		(0.25–0.73)	0.002
≦5 visits, %	0.66	(0.33–1.29)	0.220		0.21	(0.07–0.65)		0.006	0.67		(0.39–1.15)	0.148
6–10 visits, %	1.16	(0.37–3.69)	0.798		0.20	(0.02–1.65)		0.133	0.36		(0.19–0.69)	0.002
≧11 visits, %	0.66	(0.26–1.68)	0.386		1.10	(0.40–3.00)		0.855	0.25		(0.16–0.39)	<0.001

Abbreviations: RRT, renal replacement therapy; AMI, acute myocardial infarction; CCIS, Charlson Comorbidity Index; CI, confidence interval; aOR, adjusted odds ratio; CKD, chronic kidney disease; RRT, renal replacement therapy.

*Adjusted for the patients' age, gender, start of CKD care, initial RRT modality, comorbidities, Charlson comorbidity index score, socioeconomic status, urbanizations.

**Hosmer–Lemeshow goodness-of-fit statistic 0.359.

--No convergence of the estimate.

## Discussion

This study found that CKD patients with both 3 or more nephrology OPD visits in the critical care period and 11 or more visits in early care period were associated with 75% reduced composite major cardiovascular events in the 1 year after initiation of dialysis (aOR = 0.25, 95% CI = 0.16–0.39), compared with those without any visits during these two periods. Meanwhile, CKD patients with early referral were associated with 40% reduced composite major cardiovascular events only, compared with those with late referral. A total of 22.91% (261/1191) of the study population had no nephrology OPD visits in the 3 years before initiation of dialysis. These results may have implications for the future allocation of public health resources to different groups of CKD patients, many of whom receive insufficient care *via* pre-dialysis nephrology OPD visits.

Overall, 69.5% (828/1191) of the study population had at least 1 nephrology OPD visit during the critical care period, and 58.9% (701/1191) had at least 1 nephrology OPD visit during the early care period, which were patients with early referral. However, only 29.1% (346/1191) of the study population had sufficient care during both early care period and critical care period. This may partially explain why many patients who received care during the early care period nonetheless had suboptimal initiation of dialysis and unsatisfactory outcomes.

Smart et al., in a Cochrane review of 3 previous studies of CKD patients, reported that the 12-month mortality was lower in patients with early referral (within 6 months before initiation of dialysis) than in those with late referral (more than 6 months before initiation of dialysis) [11], although these 2 groups had no significant differences in ischemic heart disease and cerebrovascular disease. In agreement, the present study demonstrated that pre-dialysis care within 6 months or 6–36 months before initiation of dialysis can reduce composite major cardiovascular events. Takeshi et al. demonstrated that facility-level 1 year all-cause mortality after initiation of hemodialysis was associated with fewer nephrology visits in the 1 month before initiation of hemodialysis [16]. Jungers et al. demonstrated that longer duration of regular nephrological care in the pre-dialysis period was associated with a better long-term survival of dialysis [22]. McClellan et al. demonstrated that the absence of nephrology care in the 6 months before diagnosis of ESRD was associated with an increased risk of death [23]. Kristine et al. performed a nationwide cohort study in Denmark and reported that patients with diagnoses of diabetic nephropathy, adult polycystic kidney disease, or chronic glomerulonephritis were more likely to have received early referral [5]. Taken together with our results, this supports the view that sufficient pre-dialysis care—not simply early care or late care, but the total number of pre-dialysis nephrology OPD visits in each periods—reduces the number of composite major cardiovascular events at one–year after initiation of dialysis.

A major strength of this study is that we demonstrated the combined effect of multiple nephrology OPD visits in these two periods on composite major cardiovascular events. We calculated the total number of nephrology OPD visits, not just the presence or absence of early referral or late referral. Moreover, we expanded the outcome to composite major cardiovascular events, not just overall mortality. We also compared the annual incidence rates from Taiwan Renal Data System (TWRDS) with this study, and these rates were compatible [24].

Our study also had several limitations. First, the procedures and treatments during admission are not dated in the NHIRD, and the actual date of initiation of dialysis could be a few days lag behind than the recorded admission date. This may have led to a minor bias, but since outcomes were followed for one year, this bias would be very small due to the long follow-up period. Second, we did not analyze the effect of medication usage on patient outcomes. This topic should be examined in a future study.

## Conclusion

In conclusion, our study demonstrated that sufficient pre-dialysis nephrology OPD visits in both periods, not just early referral, can reduced the incidence of composite major vascular events by 75% during the 1-year period after initiation of dialysis. Thus, two-thirds of the CKD patients in this study could have presumably reduced their probabilities of composite major cardiovascular events if they had adequate pre-dialysis nephrology OPD visits. Medical care providers and public health policy makers may be able to improve the well-being of CKD patients who are in need of maintenance dialysis by allocating more resources to care of pre-dialysis patients and improving the education of non-nephrologist doctors and residents regarding the importance of sufficient care of these patients.

## Supporting Information

S1 Fig Algorithm of patient deposition. (DOCX)

S1 Table Mutivariable analysis of the combined effect of critical care period and early care period on one–year severe vascular events and its component. (DOCX)
