# Supplementary material for: Pre-Dialysis Visits to a Nephrology Department and Major Cardiovascular Events in Patients Undergoing Dialysis
Source: PLoS One. 2016 Feb 22;11(2):e0147508. doi: 10.1371/journal.pone.0147508 (PMC4763722; doi:10.1371/journal.pone.0147508)
Supplement: S1 Fig — (DOCX) [file pone.0147508.s001.docx]

**Supporting Information**

**S1 Fig** Algorithm of patient deposition
