# Supplementary material for: Pre-Dialysis Visits to a Nephrology Department and Major Cardiovascular Events in Patients Undergoing Dialysis
Source: PLoS One. 2016 Feb 22;11(2):e0147508. doi: 10.1371/journal.pone.0147508 (PMC4763722; doi:10.1371/journal.pone.0147508)
Supplement: S1 Table — (DOCX) [file pone.0147508.s002.docx]

**Supporting Information**

**S1 Table** Mutivariable analysis of the combined effect of critical care period and early care period on one–year severe vascular events and its components

| **aOR* of Stroke** | **Critical care period** | | | | | | | | | ****** |
| --- | --- | --- | --- | --- | --- | --- | --- | --- | --- | --- |
|  | **0 visit, *n*=363** | **95% CI** | ***P*** | **1-2 visits*, n*=155** | **95% CI** | ***P*** | **≧3 visits*, n*=673** | **95% CI** | ***P*** | **0.728** |
| **Early care period** |  |  |  |  |  |  |  |  |  |  |
| 0 visit, % | 1 |  |  | 0.33 | (0.13-0.89) | 0.029 | 0.46 | (0.22-0.94) | 0.034 |  |
| ≦5 visits, % | 0.53 | (0.20-1.45) | 0.219 | 0.65 | (0.21-2.01) | 0.454 | 0.85 | (0.43-1.69) | 0.648 |  |
| 6-10 visits, % | 2.48 | ( 0.72-8.57) | 0.153 | 0.54 | (0.06-4.56) | 0.571 | 0.31 | (0.11-0.82) | 0.018 |  |
| ≧11 visits, % | 0.82 | (0.26-2.60) | 0.730 | 0.23 | (0.03-1.80) | 0.160 | 0.26 | (0.14-0.49) | <0.001 |  |
| **aOR* of AMI** | **Critical care period** | | | | | | | | | **0.850** |
|  | **0 visit*, n*=363** | **95% CI** | ***P*** | **1-2 visits*, n*=155** | **95% CI** | ***P*** | **≧3 visits*, n*=673** | **95% CI** | ***P*** |  |
| **Early care period** |  |  |  |  |  |  |  |  |  |  |
| 0 visit, % | 1 |  |  | - |  |  | 1.40 | (0.42-4.62) | 0.583 |  |
| ≦5 visits, % | 0.61 | (0.07-5.11) | 0.648 | - |  |  | 1.14 | (0.28-4.59) | 0.853 |  |
| 6-10 visits, % | - |  |  | - |  |  | 0.82 | (0.16-4.13) | 0.810 |  |
| ≧11 visits, % | - |  |  | - |  |  | 0.70 | (0.23-2.13) | 0.529 |  |
| **aOR* of Mortality** | **Critical care period** | | | | | | | | | **0.997** |
|  | **0 visit*, n*=363** | **95% CI** | ***P*** | **1-2 visits*, n*=155** | **95% CI** | ***P*** | **≧3 visits*, n*=673** | **95% CI** | ***P*** |  |
| **Early care period** |  |  |  |  |  |  |  |  |  |  |
| 0 visit, % | 1 |  |  | 0.68 | (0.34-1.35) | 0.268 | 0.36 | (0.18-0.72) | 0.004 |  |
| ≦5 visits, % | 0.96 | (0.46-2.00) | 0.912 | 0.10 | (0.13-0.77) | 0.027 | 0.70 | (0.37-1.32) | 0.266 |  |
| 6-10 visits, % | 0.31 | ( 0.39-2.49) | 0.272 | 0.43 | (0.51-3.70) | 0.444 | 0.47 | (0.22-1.01) | 0.052 |  |
| ≧11 visits, % | 0.85 | (0.31-2.36) | 0.757 | 2.29 | (0.82-6.38) | 0.115 | 0.30 | (0.17-0.50) | <0.001 |  |
| **aOR* of composite major cardiovascular events** | **Critical care period** | | | | | | | | | **0.359** |
|  | **0 visit*, n*=363** | **95% CI** | ***P*** | **1-2 visits*, n*=155** | **95% CI** | ***P*** | **≧3 visits*, n*=673** | **95% CI** | ***P*** |  |
| **Early care period** |  |  |  |  |  |  |  |  |  |  |
| 0 visit, % | 1 |  |  | 0.42 | (0.22-0.79) | 0.007 | 0.43 | (0.25-0.73) | 0.002 |  |
| ≦5 visits, % | 0.66 | (0.33-1.29) | 0.220 | 0.21 | (0.07-0.65) | 0.006 | 0.67 | (0.39-1.15) | 0.148 |  |
| 6-10 visits, % | 1.16 | (0.37-3.69) | 0.798 | 0.20 | (0.02-1.65) | 0.133 | 0.36 | (0.19-0.69) | 0.002 |  |
| ≧11 visits, % | 0.66 | (0.26-1.68) | 0.386 | 1.10 | (0.40-3.00) | 0.855 | 0.25 | (0.16-0.39) | <0.001 |  |

Abbreviations: IQR, interquartile range; CCIS, Charlson Comorbidity Index; CKD, chronic kidney disease; HD, hemodialysis, PD, peritoneal diualysis;. aOR, adjusted odds ratio

*Adjusted for the patients' age, gender, start of CKD care, initial RRT modality, comorbidities, Charlson comorbidity index score, socioeconomic status, urbanizations.

** Hosmer–Lemeshow goodness-of-fit statistic
